# Commentary: Developing technical support and strategic dialogue at the country level to achieve primary health care-based health systems beyond the COVID-19 era

**DOI:** 10.3389/fpubh.2023.1212271

**Published:** 2023-08-25

**Authors:** Nachiket Mor

**Affiliations:** Banyan Academy of Leadership in Mental Health, Chennai, India

**Keywords:** primary care, surgical care, maternal health, health systems design, developing country

## Introduction

Cheong Chi Mo et al. (1) stress the importance of primary health care (PHC) as a central component of any health system's journey toward Universal Healthcare and discuss in detail the role that the WHO and its “large network of more than 130 health policy advisers” play in helping 115 countries build a PHC focussed pathway toward Universal Health Coverage (UHC). There is little debate about the importance of PHC within any health system. However, there are two views implicit in the position taken by Cheong Chi Mo et al. (1) which could benefit from some additional discussion: (a) if the government does not provide PHC, it does not exist; and (b) developing country governments with low health expenditures should invest in PHC as a priority. In this note, we debate both these perspectives and suggest a different approach toward prioritization that developing country governments must take.

## Availability of care

All levels of healthcare are necessary, including essential public health functions, primary care, secondary care, and tertiary care. Ideally, the state government should spend enough money so their residents can easily access all of these levels of care with no added expense. However, with limited funds, this becomes impossible for most developing country governments, and they need to focus their investments on areas of greatest need.

Data, most recently from the state of Orissa in India (2, 3), suggests the presence of a profusion of easily accessible private primary care providers, even in the remotest parts of a largely rural country like India. Extensive research on primary care by Das et al. in multiple developing countries also suggests that availability is not as much of a challenge and that, on average, the quality delivered by these primary care providers exceeds that provided by the public sector (4).

There is no similar response from the private sector in the domain of secondary care. If a high-volume emergency procedure such as a Caesarean Section (C-section) is used as a proxy for the availability of secondary care against a norm of between 10 and 20% medically necessary C-sections (5, 6), during 2019–20, most of the northern and north-eastern states in India had total (elective plus medically necessary) C-section rates well-below 10% (7). In sub-Saharan Africa, a similar situation prevails, with average C-section rates at close to 5% (8), with Nigeria showing a number as low as 2.7% (9).

## The importance of secondary care

Despite all the progress that has been made, most developing regions continue to face a high burden of disease relating to maternal and child deaths (10). In order to address this with the urgency that it needs, Nimako and Kruk (11) and Roder-DeWan et al. (12) suggest that given all the accumulating evidence, there is limited additional value to be gained from: (a) improving delivery care processes in primary care facilities (13); (b) strengthening prenatal assessment and risk stratification or movement of women needing emergency care to distant facilities since about 30% of women considered low risk develop unexpected complications during delivery (14). The way forward instead involves a comprehensive examination of the service delivery architecture, with a proposal for all mothers to give birth in or close to higher-level facilities that can provide definitive care for complications (i.e., capacity for C-Section, blood transfusion, care for sick mothers and new-borns), which offers the twin benefits of concentrating improvement efforts in fewer facilities and providing a mortality benefit through rapid access to lifesaving care (11, 15). This is also consistent with the work of Guilmoto and Dumont (16) who find that there is a strong association between increases in C-Section rates and declines in maternal mortality rates within Indian states which have C-Section rates below 20%.

Implementing these changes while presenting its own challenges is not as onerous and impossible a task in most places as might appear to be the case at first glance, even in relatively remote areas (17, 18). This may not be immediately possible for remote areas, but innovations such as hostels for birthing mothers may represent a partial solution (19, 20). The introduction of high-volume “alongside midwife-led units” or AMUs (21) located within hospitals can also be explored to address the challenges of limited obstetrician availability and to reduce the risk of the over-medicalisation of deliveries, as has been seen in several of the southern Indian states (7) and countries such as Brazil (8).

## Conclusion

The underlying message from this discussion appears clear. If a low-spending state wished to improve health outcomes for its residents, it would do well to invest in secondary care as a priority and ensure universal and high-quality availability of this care even in its more remote areas. For delivery care, it would need to build the required numbers of high-volume “alongside midwife-led units” or AMUs within these hospitals so that even as the quality of birthing care improves, the risks of moving in the direction of excessive C-Sections are minimized.

That is not to suggest that primary care is not essential but instead to recommend a different approach toward enabling and focusing it on the services it is best equipped to provide—moving away from delivery care and toward NCDs while continuing to provide ANC and PNC services. There is already a great deal of availability of such care from the private sector, and a state with limited resources could help improve the quality of services on offer instead of attempting to invest in building more of these facilities at the cost of leaving significant gaps in the availability of secondary care.

## Author contributions

The author confirms being the sole contributor of this work and has approved it for publication.

## References

[1] Cheong Chi Mo J Shah A Downey C Genay-Diliautas S Saikat S Mustafa S. Developing technical support and strategic dialogue at the country level to achieve Primary Health Care-based health systems beyond the COVID-19 era. Front Public Health. (2023) 11:1102325. 10.3389/fpubh.2023.1102325 37113176 PMC10126771

[2] Haakenstad A Kalita A Bose B Cooper JE Yip W. Catastrophic health expenditure on private sector pharmaceuticals: a cross-sectional analysis from the state of Odisha, India. Health Policy Plan. (2022) 37:872–84. 10.1093/heapol/czac035 35474539 PMC9347020

[3] Kalita A Bose B Woskie LR Haakenstad A Cooper J Yip WCM. Private pharmacies as healthcare providers in India's health system: analysis and implications for universal health coverage. BMJ Global Health (2023). 10.1136/bmjgh-2022-008903 PMC10546140 37778756

[4] Das J Woskie L Rajbhandari R Abbasi K Jha A. Rethinking assumptions about delivery of healthcare: implications for universal health coverage. BMJ. (2018) 361:k1716. 10.1136/bmj.k1716 29784870 PMC5961312

[5] Betran AP Torloni MR Zhang JJ Gulmezoglu AM Aleem HA Althabe F. WHO statement on caesarean section rates. BJOG. (2015) 123:667–70. 10.1111/1471-0528.13526 26681211 PMC5034743

[6] Molina G Weiser TG Lipsitz SR Esquivel MM Uribe-Leitz T Azad T. Relationship between cesarean delivery rate and maternal and neonatal mortality. JAMA. (2015) 314:2263. 10.1001/jama.2015.15553 26624825

[7] GIL (2021),. NFHS Policy Tracker for Districts. Geographic Insights Lab, Harvard University. Available online at: https://geographicinsights.iq.harvard.edu/nfhs-tracker-districts (accessed December 24, 2021).

[8] Betran AP Ye J Moller A-B Souza JP Zhang J. Trends and projections of caesarean section rates: global and regional estimates. BMJ Global Health. (2021) 6:e005671. 10.1136/bmjgh-2021-005671 34130991 PMC8208001

[9] Berglundh S Benova L Olisaekee G Hanson C. Caesarean section rate in Nigeria between 2013 and 2018 by obstetric risk and socio-economic status. Trop Med Int Health. (2021) 26:775–88. 10.1111/tmi.13579 33780090

[10] Emadi M Delavari S Bayati M. Global socioeconomic inequality in the burden of communicable and non-communicable diseases and injuries: an analysis on global burden of disease study 2019. BMC Public Health. (2021) 21:1771. 10.1186/s12889-021-11793-7 34583668 PMC8480106

[11] Nimako K Kruk ME. Seizing the moment to rethink health systems. Lancet Global Health. (2021) 9:e1758–62. 10.1016/S2214-109X(21)00356-9 34506770 PMC8423432

[12] Roder-DeWan S Nimako K Twum-Danso NAY Amatya A Langer A Kruk M. Health system redesign for maternal and newborn survival: rethinking care models to close the global equity gap. BMJ Global Health. (2020) 5:e002539. 10.1136/bmjgh-2020-002539 33055093 PMC7559116

[13] Semrau KEA Hirschhorn LR Marx Delaney M Singh VP Saurastri R Sharma N. Outcomes of a coaching-based WHO safe childbirth checklist program in India. N Engl J Med. (2017) 377:2313–24. 10.1056/NEJMoa1701075 29236628 PMC5672590

[14] Danilack VA Nunes AP Phipps MG. Unexpected complications of low-risk pregnancies in the United States. Am J Obstetr Gynecol. (2015) 212:809.e1–e6. 10.1016/j.ajog.2015.03.038 26042957 PMC4728153

[15] Goldenberg RL McClure EM. Improving birth outcomes in low- and middle-income countries. N Engl J Med. (2017) 377:2387–8. 10.1056/NEJMe1713831 29236634

[16] Guilmoto CZ Dumont A. Trends, regional variations, and socioeconomic disparities in Cesarean Births in India, 2010-2016. JAMA Netw Open. (2019) 2:e190526. 10.1001/jamanetworkopen.2019.0526 30901040 PMC6583298

[17] Gage AD Carnes F Blossom J Aluvaala J Amatya A Mahat K. In low- and middle-income countries, is delivery in high-quality obstetric facilities geographically feasible? Health Aff. (2019) 38:1576–84. 10.1377/hlthaff.2018.05397 31479351

[18] Nimako K Gage A Benski C Roder-DeWan S Ali K Kandie C. Health system redesign to shift to hospital delivery for maternal and newborn survival: feasibility assessment in Kakamega County, Kenya. Global Health Sci Pract. (2021) 9:1000–10. 10.9745/GHSP-D-20-00684 34933993 PMC8691889

[19] Bayya V,. ITDA Starts Setting Up Hostels for Expectant Mothers in Tribal Areas. Times of India (2019). Available online at: https://timesofindia.indiatimes.com/city/visakhapatnam/itda-starts-setting-uphostels-for-expectant-mothers-in-tribalareas/articleshow/69403149.cms (accessed December 03, 2021).

[20] Nabirye J, Nabiryo, M, Awor, P,. The Mothers' Waiting Hostel in Bwindi. (2018). Available online at: https://socialinnovationinhealth.org/downloads/Case_Studies/BCU_case_study.pdf (accessed December 03, 2021).

[21] Rayment J McCourt C Rance S Sandall J. What makes alongside midwifery-led units work? Lessons from a national research project. Pract Midwife. (2015) 18:31–3. 26320335

